# Benign Recurrent Sixth (Abducens) Nerve Palsy following Measles-Mumps-Rubella Vaccination

**DOI:** 10.1155/2015/734516

**Published:** 2015-07-14

**Authors:** Areti Bourtoulamaiou, Sohraab Yadav, Harish Nayak

**Affiliations:** Alder Hey Children's Hospital, Eaton Road, Liverpool, Merseyside L12 2AP, UK

## Abstract

Benign, isolated, recurrent sixth nerve palsy is rare in children. It may be associated with febrile viral illness and vaccination in exceptional circumstances although this is a diagnosis of exclusion. Here, we present the case of a 2-year-old Caucasian girl who developed recurrent 6th nerve palsy following vaccination with the measles-mumps-rubella (MMR) vaccine. No underlying pathology was identified following extensive investigations and followup. There is limited data available on the pathophysiology of vaccination-related nerve palsies. As with all previous reports of cranial nerve palsies following vaccination, there was complete resolution in this case. Long term followup with repeated physical examination and investigations is warranted to avoid missing severe pathology and operating unnecessarily.

## 1. Introduction

Palsy of the sixth (abducens) cranial nerve results in an incomitant strabismus and abduction deficit. Sixth nerve palsy is far less frequent in children and common causes include trauma, raised intracranial pressure, and neoplasm necessitating thorough investigation [1].

We present a case of a two-year-old girl who was referred to our department with a sudden onset of left eye esotropia followed by right eye esotropia one week after receiving the measles-mumps-rubella (MMR) vaccination.

## 2. Case Presentation

A two-year-old child attended the eye casualty with her parents. She had a three-week history of sudden onset strabismus. Initial orthoptic examination revealed a primary position right eye esotropia measuring 30–35 prism diopters for near fixation, with a right abduction deficit, lateral incomitance, and no palpebral fissure changes.

Her ophthalmic and neurological examination was otherwise normal with no symptoms or signs of any other cranial nerve involvement. Visual acuity was normal and there was no significant refractive error on cycloplegic refraction for her age (+2.25 sph/+1.50 cyl × 180° RE and +2.25 sph/+1.25 cyl × 180° LE). An urgent CT scan was ordered which was normal. A diagnosis of sixth nerve palsy was made and the child was followed up closely by the paediatric neurology and ophthalmology team.

One week later, there was a slight improvement in the degree of esotropia but the patient had developed a right face turn on fixation. Left eye patching was commenced for 12 hours per day, 3 days per week. An MRI scan of the brain was also undertaken on the day and was reported as normal.

Complete and spontaneous resolution was noted over 8 weeks after initial presentation and patching was cut down by half for four more weeks and then stopped.

Seven months later, the child returned with a recurrent right eye esotropia measuring approximately 12 prism diopters, with a compensatory face turn to the right. Investigations including neuroimaging and blood tests were again normal and the palsy resolved completely and spontaneously within one week. One more episode of recurrent right eye small abduction deficit developed four weeks later, followed by spontaneous resolution within two weeks. During this period of time, the patient was systemically fit and well. The child remains under regular followup in the ophthalmology department.

## 3. Discussion

Isolated sixth nerve palsies in children may be congenital or acquired. Common acquired causes include postviral, trauma, and idiopathic ones [2–4]. Viral causes have most commonly been attributed to the herpes family such as herpes zoster, Epstein Barr virus, HSV-1, and Cytomegalovirus [5, 6]. Malignancy may occasionally present with a chronic isolated sixth nerve palsy [7]. For this reason, many authorities recommend the use of neuroimaging in all children with acute and chronic sixth nerve palsies [8]. Lumbar puncture with cerebrospinal fluid analysis for viral PCR may aid diagnosis although this was not performed in our patient as it was deemed too invasive. Further investigations that may be warranted based on a suggestive history are electrodiagnostic tests, such as visual evoked potentials and electroretinography, and serum antiganglioside antibodies for association with Guillain-Barre syndrome [9].

Recurrent 6th nerve palsy in a child following different live attenuated vaccines has been reported before [3, 10–12]. In all cases, complete spontaneous resolution has occurred but this may vary from days to months [13]. Failure to improve suggests more serious intracranial pathology [7]. The MMR vaccine in particular has also been associated with other neurologic disorders [14] but its overall safety and efficacy have been demonstrated [15].

The underlying pathophysiology behind vaccination-related cranial nerve palsies has still not been fully discerned. Postulated mechanisms include immune mediated damage resulting in demyelination or localised reduction in blood flow to the nerve [3].

In our case, thorough investigation was carried out before commencing treatment. Patching was the chosen treatment modality to prevent amblyopia and allow for binocular visual function in the long term. Children do not complain of diplopia but develop a compensatory head posture as what happened in our case. Parents are advised not to discourage their child when such a face turn is noted, as it is important for the maintenance of binocular single vision. Other treatment options such as alternate patching, prism therapy, strabismus surgery, and/or botulinum toxin injection should be considered if no improvement is noted.

Long term observation is vital in children with recurrent sixth nerve palsy and the treatment plan should be based on the underlying cause. Vaccination-induced sixth nerve palsy is always a diagnosis of exclusion given the more serious and life-threatening alternative causes.
